# Development and validation of a nomogram to predict medication risk based on a knowledge, attitude and practice (KAP) survey of residents in Shanxi Province, China

**DOI:** 10.3389/fphar.2024.1302274

**Published:** 2024-04-17

**Authors:** Da-shuai Xie, Xue-hu Xie, Li-hua Yang, Na Li, Xiao Zhang, Yi-tong Xie, Wei Yang, Yao-jun Ning, Jun Xie, Xiao-jun Cheng, Shao-jun Duan, Shi-wei Wang, Li-hong Hao, Ping Shi

**Affiliations:** ^1^ Department of Pharmacy, Yuncheng Central Hospital Affiliated to Shanxi Medical University, Yuncheng, China; ^2^ National Drug Clinical Trial Institution, Shanxi Province Hospital of Traditional Chinese Medicine, Taiyuan, China; ^3^ Department of Pharmacy, Jincheng People’s Hospital, Jincheng, China; ^4^ Department of Gynecologic, Yuncheng Central Hospital Affiliated to Shanxi Medical University, Yuncheng, China; ^5^ Department of Pharmacy, Shanxi Province Hospital of Traditional Chinese Medicine, Taiyuan, China; ^6^ Department of Pharmacy, Yangquan Coal Industry (Group) General Hospital, Yangquan, China

**Keywords:** KAP, medication, factor, nomogram, resident, China

## Abstract

**Objective:**

Unsafe medication practices and medication errors are a major cause of harm in healthcare systems around the world. This study aimed to explore the factors that influence the risk of medication and provide medication risk evaluation model for adults in Shanxi province, China.

**Methods:**

The data was obtained from the provincial questionnaire from May to December 2022, relying on the random distribution of questionnaires and online questionnaires by four hospitals in Shanxi Province. Multiple linear regression analysis was used to explore the factors affecting the KAP score of residents. Univariate and multivariate logistic regression was used to determine the independent risk factors, and the nomogram was verified by receiver operating characteristic curve, calibration and decision curve analysis.

**Results:**

A total of 3,388 questionnaires were collected, including 3,272 valid questionnaires. The average scores of drugs KAP were 63.2 ± 23.04, 33.05 ± 9.60, 23.67 ± 6.75 and 33.16 ± 10.87, respectively. On the evaluation criteria of the questionnaire, knowledge was scored “fair”, attitude and practice were scored “good”. Sex, monthly income, place of residence, insurance status, education level, and employment were regarded as independent risk factors for medication and a nomogram was established by them.

**Conclusion:**

Males, low-income, and low-educated people are important factors affecting the risk of medication. The application of the model can help residents understand the risk of their own medication behavior and reduce the harm of medication.

## 1 Introduction

Unsafe medication practices and medication errors are a leading cause of injury and avoidable harm in healthcare systems from all over the world (Glavin, 2010; Rishoej et al., 2018; Mulac et al., 2022). Adverse drug events lead to 2.5 million hospitalizations in China every year, and about 190,000 of them result in death (Fan et al., 2018). According to a global estimate, medication errors cost $42 billion a year. To address this issue, WHO has launched the third Global Patient Safety Challenge with the theme of “Medication Without Harm” (World Health Organization, 2017). It is essential to raise public awareness of the safety risks associated with medication use and the need for safer medication practices.

Self-medication is the practice of using drugs to treat common diseases and health problems without consulting a doctor or a pharmacist (Ruiz, 2010). It is very common in China, where people can easily access drug information through various media channels. Self-medication can help protect health and save medical costs, but it can also pose significant health risks (Hughes et al., 2001; Stosic et al., 2011). Many surveys and studies have shown that Chinese residents often use drugs improperly because of factors such as socioeconomic level, cognitive level, traditional culture, personal experiences, limited medication knowledge, and false advertising. Some examples of improper drug use are blind medication, not following drug instructions, wrong administration methods, arbitrary changes in dosage and duration, and using health supplements as drugs. Irrational drug use can lead to serious health problems and even life-threatening consequences for Chinese residents (Hu et al., 2021; Wu et al., 2021).

Shanxi is a significant resource province in China that supplies coal power resources for 24 provinces across the China in 2022. As the economy grows, the people here are becoming more aware of health issues and the healthcare costs are rising every year (He et al., 2022). To ensure the health of residents, we as medical workers need to examine and understand their medication situation and the potential risks involved in the medication process, especially regarding self-medication. The KAP model comprises three components: knowledge, attitude, and practice. It is a common model that explains how residents’ knowledge, beliefs, and behaviors affect their health (Asante et al., 2023). By imparting knowledge, fostering confidence, and ultimately modifying their behavior, the KAP model has been widely applied in healthcare activities such as preventive healthcare, intervention evaluation, and medication behavior risk assessment (Chaudhary et al., 2023; Failoc-Rojas et al., 2023). The KAP model can help us explore the relationships among knowledge, attitude, and practice behavior of health or the associations between basic resident information and KAP (Dopelt et al., 2023; Liao and Yang, 2023).

The main challenge at present is that the residents are unaware of their own medication cognitive level, and the doctor or pharmacist cannot devote much time to each patient in real life. It is impractical for the residents to spend a lot of time evaluating their medication every time they see a doctor. Therefore, it is essential to develop a simple and practical predictive model to assess the risks of self-medication for the residents. Multivariate logistic regression analysis is a multivariate analysis method that examines the dependent variable as binary observations and influencing factors. It is widely used in data mining, economic forecasting, disease risk factor analysis, and other fields (Sheikh et al., 2022; Allen et al., 2023; Yuan et al., 2023). The nomogram is a graphical representation of a logistic regression model that visualizes a prediction (Olofsson Bagge et al., 2020; Serenari et al., 2020; Raghav et al., 2021). However, there is no report on using a nomogram-based model to evaluate the risks of self-medication.

**FIGURE 1:**
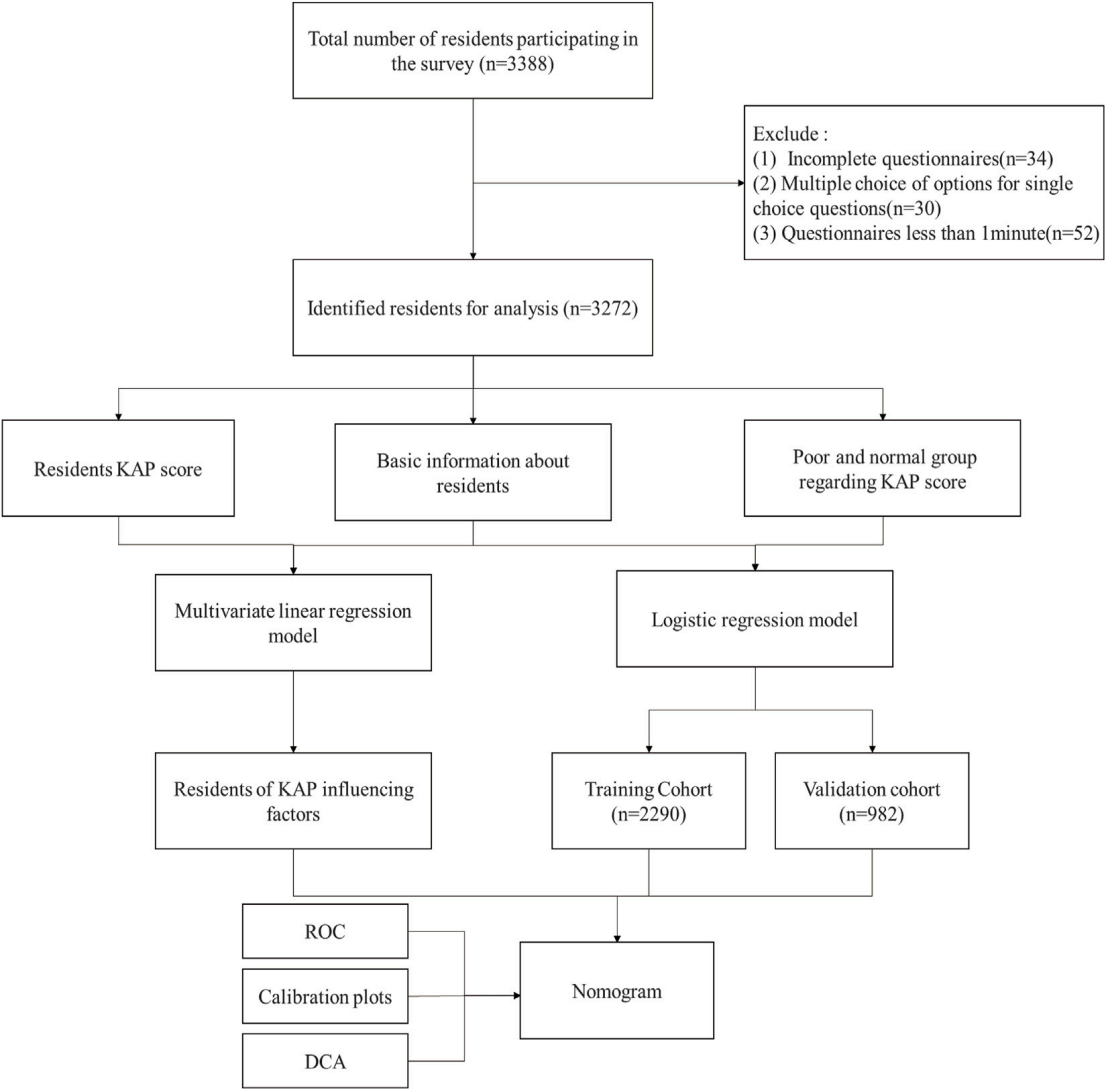
The flow chart of the data selection and analysis process.

Thus, the objective of this study is aimed to build a nomogram based on multiple logistic regression after the KAP survey and to predict the risk profiles for medication practice (Figure 1).

## 2 Methods

### 2.1 Study design and patient population

This regional survey was conducted in Shanxi Province, where four major cities—Taiyuan, Yuncheng, Jincheng, and Yangquan—were selected for investigation and research. The Yangquan Coal Industry (Group) General Hospital, Shanxi Provincial Hospital of Traditional Chinese Medicine, Jincheng People’s Hospital, and Yuncheng Central Hospital are located in the northeast, central, northwest, and southwest of Shanxi Province, respectively. Each institution participated in the trial, secured approval from their respective ethics committees.

The survey time of the questionnaire was from May 2022 to December 2022, and the survey was carried out by means of offline and online questionnaires for residents aged 19 years and older who voluntarily participated in the survey.

### 2.2 Questionnaire information

The questionnaire is developed by the Science and Technology Development Center of the Chinese Pharmaceutical Association, and the content includes the following items:

a. Basic information of residents, including sex, age, monthly income, place of residence, health insurance status, education level, employment status, and occupation (eight questions);
b. Residents’ medication knowledge, including the basic knowledge of self-medication and their opinions on drugs and drug use (twenty-eight questions);
c. Residents’ attitude towards medication education, including the frequency and need of attending medicinal lectures or activities (eleven questions);
d. Survey of residents’ medication practice, including good medication practice (seven questions) and bad medication practice (seventeen questions).

Refer to the Likert 5-level rating scale, and quantify the actual frequency of behavior and the degree of approval: one point for strong disapproval, two points for disapproval, three points for neutral, four points for approval, five points for strong approval, and six points for unclear. The higher score on medication knowledge questions, the higher risk level; the lower your score on medication attitude questions, the higher your risk level; for medication practice questions, a lower score on good practices and a higher score on bad practices indicate a higher risk level. The resident rating scale is shown in Table 1.

**TABLE 1:** Resident KAP score scale.

Score grading	Good	Fair	Poor
Knowledge score	28∼56	57∼84	>84
Attitude score	>33	23∼33	11∼22
Good Practice score	>21	15∼21	7∼14
Bad Practice score	17∼34	35∼51	>51

Before conducting the survey, the investigators received unified training, including introduce the content and purpose of the survey to the potential respondents and ensure that the participation was voluntary. In order to ensure the validity and reliability of the survey, it was stipulated that one valid questionnaire can be submitted only once per IP address. Any questionnaires that were less than 1 min, incomplete, or multiple-choice were excluded.

### 2.3 Statistical analysis

The survey data were recorded in Excel 2019 and analyzed by the SPSS version 22.0 and R version 4.2.3 for Windows. Categorical variables were expressed in frequency and percentage, the normal distribution test was carried out by the Shapiro-wilk method for continuous variables, and the x ± SD, was used for the normal distribution, the between-group comparison was represented by independent sample *t*-test or one-way ANOVA, the non-normal distribution was represented by M (Q1, Q3), and the between-group comparison was represented by the Mann-Whitney U test. Multivariate linear regression models were used to analyze the correlation between basic resident information and the scores of KAP, and to find the influencing factors of the resident KAP.

In this study, the “poor” score in KAP is defined as the risky mediation practice, the rest as normal practice. The KAP data of residents were randomly subdivided into a training cohort of 70% and a validation cohort of 30%. The logistic regression model was used for univariable and multivariable analysis to assess the independent risk factors for resident medication practice, the odds ratio (OR) and 95% confidence interval (95% CI) were calculated. Significant influencing factors were counted in the multivariable analysis and used to build a new nomogram for predicting the risk of resident medication practice. The accuracy and reliability of the nomogram were verified by the validation cohort. The receiver operating characteristic (ROC) curve was constructed and the predictive accuracy of the nomogram was evaluated by the area under the curve (AUC). The calibration plots were used to verify the accuracy of the predictions of the nomogram. Decision curve analysis (DCA) was used to evaluate the clinical significance of the nomogram (Vickers et al., 2008). Statistical significance was defined as *p* < 0.05. When the same dataset is analyzed multiple times, the *p*-values should be adjusted using the Benjamini-Hochberg method (Author Anonymous, 2023).

## 3 Results

### 3.1 Basic information of subjects

A total of 3,388 resident questionnaires were collected and 3,272 of them were included in the analysis, accounting for 96.57% of the total (Table 2). Each institution that participated in the trial collected 1,421; 851; 500; and 500 valid questionnaires, respectively. Of these, 2,188 were obtained through online methods, and 1,084 were collected offline. The survey participants included 1,240 males and 2,032 females, mostly young (19–34) or middle-aged (35–49) residents (77.20%). Most residents (64.18%) had an income range between 1,000–2000 or 2000–4,000 yuan, and lived in urban areas (75.37%). Most had basic social medical insurance (70.90%), and had mainly junior college or bachelor’s degree education (59.90%). Most residents were employed (72.80%), and mainly worked as factory or healthcare workers (46.27%).

**TABLE 2:** Key demographics of participants.

Characteristics	NO.	Percentage (%)	Knowledge		Attitude		Good practice		Bad practice	
			x±SD	*p*	x±SD	*p*	x±SD	*p*	x±SD	*p*
Sex										
Male	1,240	37.90	66.74 ± 25.24	0.00**	32.53 ± 9.94	0.02*	23.34 ± 6.90	0.03*	33.77 ± 11.47	0.01*
Female	2032	62.10	61.04 ± 21.31		33.37 ± 9.38		23.87 ± 6.64		32.78 ± 10.47	
Age, years										
19–34	1,351	41.29	60.13 ± 21.36	0.00**	32.75 ± 9.54	0.01*	22.78 ± 6.71	0.00**	32.54 ± 10.27	0.00**
35–49	1,175	35.91	62.85 ± 23.65		32.87 ± 9.00		24.08 ± 6.82		32.76 ± 10.43	
50–64	580	17.73	67.70 ± 23.90		34.33 ± 10.87		24.58 ± 6.44		34.23 ± 12.11	
65 and older	166	5.07	74.93 ± 22.75		32.33 ± 9.16		24.74 ± 6.74		37.22 ± 12.84	
Monthly income (CNY)										
Below 1,000	362	11.06	70.54 ± 27.25	0.00**	33.31 ± 11.32	0.39	23.53 ± 7.2	0.49	35.10 ± 12.93	0.00**
1,000–2000	784	23.96	66.92 ± 24.17		33.25 ± 10.06		23.90 ± 7.45		34.89 ± 12.07	
2000–4,000	1,316	40.22	61.07 ± 21.49		33.22 ± 9.19		23.47 ± 6.13		31.76 ± 9.38	
4,000–6,000	565	17.27	59.68 ± 18.69		32.40 ± 8.01		23.70 ± 6.33		31.83 ± 8.60	
Above 6,000	245	7.49	59.98 ± 25.67		32.60 ± 10.81		24.15 ± 7.72		35.31 ± 13.72	
Place of residence										
Urban areas	2,466	75.37	61.25 ± 21.55	0.00**	32.83 ± 9.40	0.03*	23.89 ± 6.56	0.00**	32.78 ± 10.06	0.00**
Rural areas	806	24.63	69.17 ± 26.23		33.72 ± 10.17		22.99 ± 7.26		34.30 ± 12.96	
Health insurance coverage										
Basic social medical insurance	2,320	70.90	61.73 ± 22.06	0.00**	32.93 ± 9.18	0.57	23.72 ± 6.45	0.00**	32.42 ± 10.06	0.00**
Commercial insurance	114	3.48	69.83 ± 28.59		33.96 ± 10.55		24.53 ± 8.21		38.33 ± 14.37	
Out-of-pocket medical care	205	6.27	73.4 ± 27.59		33.58 ± 11.64		22.81 ± 7.14		35.92 ± 13.44	
Publicly-funded medical care	418	12.78	63.12 ± 21.28		33.40 ± 9.82		24.2 ± 6.70		33.59 ± 10.64	
Others	215	6.57	65.95 ± 25.44		32.63 ± 10.89		22.41 ± 8.39		34.92 ± 13.23	
Education level										
Graduate student	278	8.50	54.27 ± 22.78	0.00**	32.71 ± 9.31	0.03*	23.86 ± 7.07	0.00**	33.38 ± 10.78	0.00**
Bachelor	1,223	37.38	56.37 ± 17.98		32.68 ± 8.64		23.24 ± 6.41		31.66 ± 8.48	
Junior college	737	22.52	62.42 ± 20.73		32.61 ± 9.68		23.03 ± 6.32		31.82 ± 9.61	
Technical secondary or high school	551	16.84	70.85 ± 22.14		33.60 ± 9.54		24.43 ± 6.62		34.04 ± 12.04	
Middle school	393	12.01	77.11 ± 27.18		34.25 ± 11.55		24.69 ± 7.81		36.99 ± 14.22	
Primary school	90	2.75	82.47 ± 33.71		34.06 ± 12.55		25.04 ± 8.31		41.64 ± 15.81	
Employment										
Currently employed	2,382	72.80	59.88 ± 20.66	0.00**	32.83 ± 9.11	0.06	23.43 ± 6.58	0.01*	32.23 ± 9.85	0.00**
Retired	407	12.44	71.39 ± 24.03		34.02 ± 10.07		24.48 ± 6.72		35.06 ± 12.68	
Unemployed or jobless	483	14.76	72.66 ± 28.40		33.30 ± 11.36		24.15 ± 7.46		36.14 ± 13.07	
Occupation										
Factory workers	625	19.10	70.42 ± 24.10	0.00**	32.45 ± 9.79	0.09	23.5 ± 6.49	0.67	33.84 ± 11.76	0.00**
Company employees	325	9.93	64.28 ± 22.52		32.2 ± 10.04		23.6 ± 6.93		33.84 ± 10.73	
Government cadres	231	7.06	63.93 ± 18.98		33.73 ± 10.51		23.76 ± 6.19		32.92 ± 10.00	
Healthcare workers	889	27.17	50.53 ± 13.73		33.51 ± 8.00		23.69 ± 6.47		30.02 ± 8.03	
Teachers	169	5.17	60.84 ± 20.34		31.65 ± 7.82		23.62 ± 6.24		33.12 ± 8.46	
Business managers	73	2.23	64.59 ± 21.72		33.58 ± 8.98		23.51 ± 6.42		33.77 ± 7.08	
Freelancers	295	9.02	73.88 ± 26.28		33.23 ± 10.73		23.80 ± 7.62		35.98 ± 12.83	
Students	175	5.35	62.90 ± 22.54		33.88 ± 11.06		22.78 ± 6.79		33.72 ± 10.96	
Others	490	14.98	70.20 ± 25.97		33.23 ± 10.55		24.12 ± 7.31		35.65 ± 13.02	
Total	3,272		63.20 ± 23.04		33.05 ± 9.60		23.67 ± 6.75		33.16 ± 10.87	

*p< 0.05; **p< 0.01.

### 3.2 KAP scores of medications among residents

Residents’ medication knowledge had an average score of 63.2 ± 23.04, which was rated as “fair” based on the quantitative scoring criteria. The cognitive scores of drugs and their use varied significantly by sex, age, monthly income, place of residence, health insurance coverage, education level, employment status and occupation of the respondents (*p* < 0.05). The average score of residents’ medication attitude was 33.05 ± 9.60, and the overall score was “good” according to the quantitative criteria of the score. The scores of contact frequency and attitude towards drug education activities differed significantly by sex, age, place of residence and education level of the respondents (*p* < 0.05). Residents’ good practice had an average score of 23.67 ± 6.75, which was rated as “good” based on the quantitative scoring criteria. There were significant differences in the score of good practice among residents of different sex, age, place of residence, Health insurance coverage, education level and employment status (*p* < 0.05). Residents’ bad practice had an average score of 33.16 ± 10.87, which was rated as “good” based on the quantitative scoring criteria, indicating a low risk. The score for bad practice in drug use differed significantly by sex, age, monthly income, place of residence, health insurance coverage, education level, employment status and occupation of the respondents (*p* < 0.05).

Based on the scoring criteria, we grouped the error-prone questions in the KAP survey of medicines (Table 3) and assessed the risks of residents in relation to antibiotic use, drug storage, disposal of expired drugs, and adjustment of the drug dose. Meanwhile, the traditional medical science popularization and education work, such as organizing lectures and consultations, should also adapt to the changing times, shifting from offline to online platforms, such as the Internet, WeChat public accounts and other channels, which will be more effective.

**TABLE 3:** Questions with a low accuracy rate in the resident KAP questionnaire.

KAP	Issue	Correct or approval rate (%)
Knowledge	1.As long as you do not abuse antimicrobials, you will not develop resistance	54.46
	2.Medicines that run out of use should be stored in the refrigerator as much as possible	56.82
	3.When buying drugs, the price does not matter, the key is good efficacy	37.56
Attitude	1.In my community, I listened to a lecture on the knowledge of rational drug use in the community	6.45
	2.Listen to lectures on rational drug use in hospitals or community health service centers	10.27
	3.Participate in pharmacist community or street counseling	9.29
Good Practice	1.Go to the pharmacy with your doctor’s prescription to buy prescription drugs	20.90
	2.Regular inspections of medicines stored at home	29.89
	3.Check the drug approval number on the drug packaging before taking the medicine	27.81
Bad Practice	1.Discard expired medicines at home in the trash	37.59
	2.When taking medication, you will consider your physical condition every time	38.02
	3.After the condition improves, reduce the dose or stop the drug on your own	67.76

### 3.3 Multiple linear regression analysis

The KAP scores were used as dependent variables, and basic information of residents were the independent variables. A multiple linear regression model was used to analyze the relationship (Table 4). The results revealed that sex, place of residence, education level and employment status were the factors affecting medication knowledge; Sex was the only factor influencing attitudes toward medication; Sex, age, place of residence, health insurance coverage, and education level were the factors influencing good practices toward medication; Bad practices of medication was influenced by sex, health insurance coverage, education level, and employment status.

**TABLE 4:** Factors influencing the KAP score of medication.

Characteristics	Knowledge			Attitude			Good practice			Bad practice		
	*β*	*T*	*p*	*β*	*T*	*p*	*β*	*T*	*p*	*β*	*T*	*p*
Sex	−0.10	−5.92	0.00**	0.05	2.61	0.01*	0.05	2.87	0.00**	−0.04	−2.12	0.03*
Age, years	0.01	0.56	0.57	0.03	1.47	0.14	0.08	3.50	0.00**	0.02	0.74	0.46
Monthly income	−0.04	−1.96	0.05	0.00	−0.03	0.98	0.03	1.60	0.11	0.01	0.45	0.65
Place of residence	0.04	2.36	0.02*	0.03	1.71	0.09	−0.07	−3.55	0.00**	−0.01	−0.31	0.76
Health insurance coverage	0.01	0.33	0.74	−0.01	−0.28	0.78	−0.04	−2.16	0.03*	0.05	2.73	0.01*
Education level	0.28	13.71	0.00**	0.04	1.73	0.08	0.06	2.84	0.01*	0.12	5.40	0.00**
Employment	0.09	4.22	0.00**	−0.01	−0.58	0.56	0.03	1.41	0.16	0.07	3.13	0.00**
Occupation	−0.04	−1.99	0.05	0.02	0.85	0.40	0.03	1.37	0.17	0.04	1.84	0.07
F	68.48			2.70			8.97			16.14		
Adjusted R^2^	0.14			0.00			0.02			0.04		
*p*	0.00**			0.01*			0.00**			0.00**		

*p< 0.05; **p< 0.01.

### 3.4 Univariate and multivariate analysis

Univariate analysis showed that sex, age, monthly income, place of residence, health insurance coverage, education level, employment status, and occupation were significant risk factors for KAP of medication. We adjusted the related risk factors with *p* < 0.05 in univariate analyses for the multivariate analysis. The multivariate analysis revealed that only sex, monthly income, health insurance coverage, education level and employment status remained as independent risk factors for KAP of medication (Table 5).

**TABLE 5:** Univariable and multivariable logistic analysis of medication-related risks in the training cohort.

Characteristics	Univariate analysis			Multivariate analysis		
	OR	CI	*p*	OR	CI	*p*
Sex						
Male	Reference			Reference		
Female	0.64	0.55–0.74	0.00**	0.64	0.53–0.76	0.00**
Age, years						
19–34	Reference			Reference		
35–49	1.12	0.94–1.34	0.24	1.02	0.83–1.25	0.97
50–64	1.70	1.38–2.08	0.00**	1.00	0.74–1.34	0.98
65 and older	2.21	1.59–3.08	0.00**	0.78	0.49–1.25	0.53
Monthly income						
Below 1,000	Reference			Reference		
1,000–2000	0.77	0.60–0.99	0.06	0.90	0.65–1.25	0.75
2000–4,000	0.40	0.32–0.52	0.00**	0.59	0.42–0.82	0.01*
4,000–6,000	0.30	0.22–0.4	0.00**	0.46	0.31–0.68	0.00**
Above 6,000	0.55	0.39–0.77	0.00**	0.71	0.46–1.11	0.28
Place of residence						
Urban areas	Reference			Reference		
Rural areas	1.95	1.65–2.30	0.00**	1.08	0.88–1.33	0.75
Health insurance coverage						
Basic social medical insurance	Reference			Reference		
Commercial insurance	2.71	1.85–3.95	0.00**	2.16	1.44–3.25	0.00**
Out-of-pocket medical care	2.52	1.89–3.36	0.00**	1.59	1.15–2.19	0.02*
Publicly-funded medical care	0.99	0.78–1.25	0.93	0.97	0.75–1.26	0.97
Others	3.75	2.82–4.99	0.00**	2.62	1.90–3.60	0.00**
Education level						
Graduate student	Reference			Reference		
Bachelor	0.81	0.60–1.10	0.22	0.80	0.57–1.11	0.34
Junior college	1.21	0.88–1.66	0.28	0.92	0.65–1.31	0.86
Technical secondary or high school	1.61	1.17–2.23	0.01*	0.95	0.65–1.39	0.97
Middle school	3.89	2.77–5.45	0.00**	1.68	1.10–2.56	0.04*
Primary school	5.60	3.36–9.33	0.00**	1.87	1.03–3.41	0.09
Employment						
Currently employed	Reference			Reference		
Retired	2.39	1.92–2.97	0.00**	1.94	1.40–2.67	0.00**
Unemployed or jobless	3.34	2.73–4.09	0.00**	1.49	1.11–2.01	0.02*
Occupation						
Factory workers	Reference			Reference		
Company employees	0.84	0.63–1.12	0.28	0.98	0.72–1.35	0.97
Government cadres	0.79	0.58–1.10	0.22	1.12	0.79–1.59	0.75
Healthcare workers	0.28	0.22–0.36	0.00**	0.42	0.32–0.55	0.00**
Teachers	0.50	0.34–0.75	0.00**	0.75	0.49–1.15	0.34
Business managers	0.88	0.52–1.47	0.64	0.98	0.56–1.71	0.97
Freelancers	1.45	1.09–1.92	0.01*	0.88	0.63–1.24	0.75
Students	0.89	0.62–1.27	0.55	0.52	0.32–0.84	0.02*
Others	1.53	1.20–1.95	0.00**	1.03	0.77–1.37	0.97

*p< 0.05; **p< 0.01. The *p*-values in the tables were the adjusted values, which were adjusted using the Benjamini-Hochberg method.

### 3.5 Construction and validation of the nomogram

**FIGURE 2:**
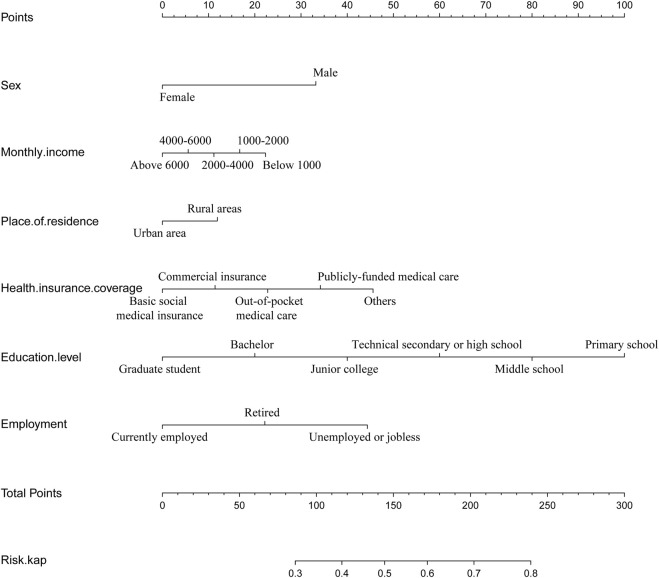
The nomogram to predict medication risks.

**FIGURE 3:**
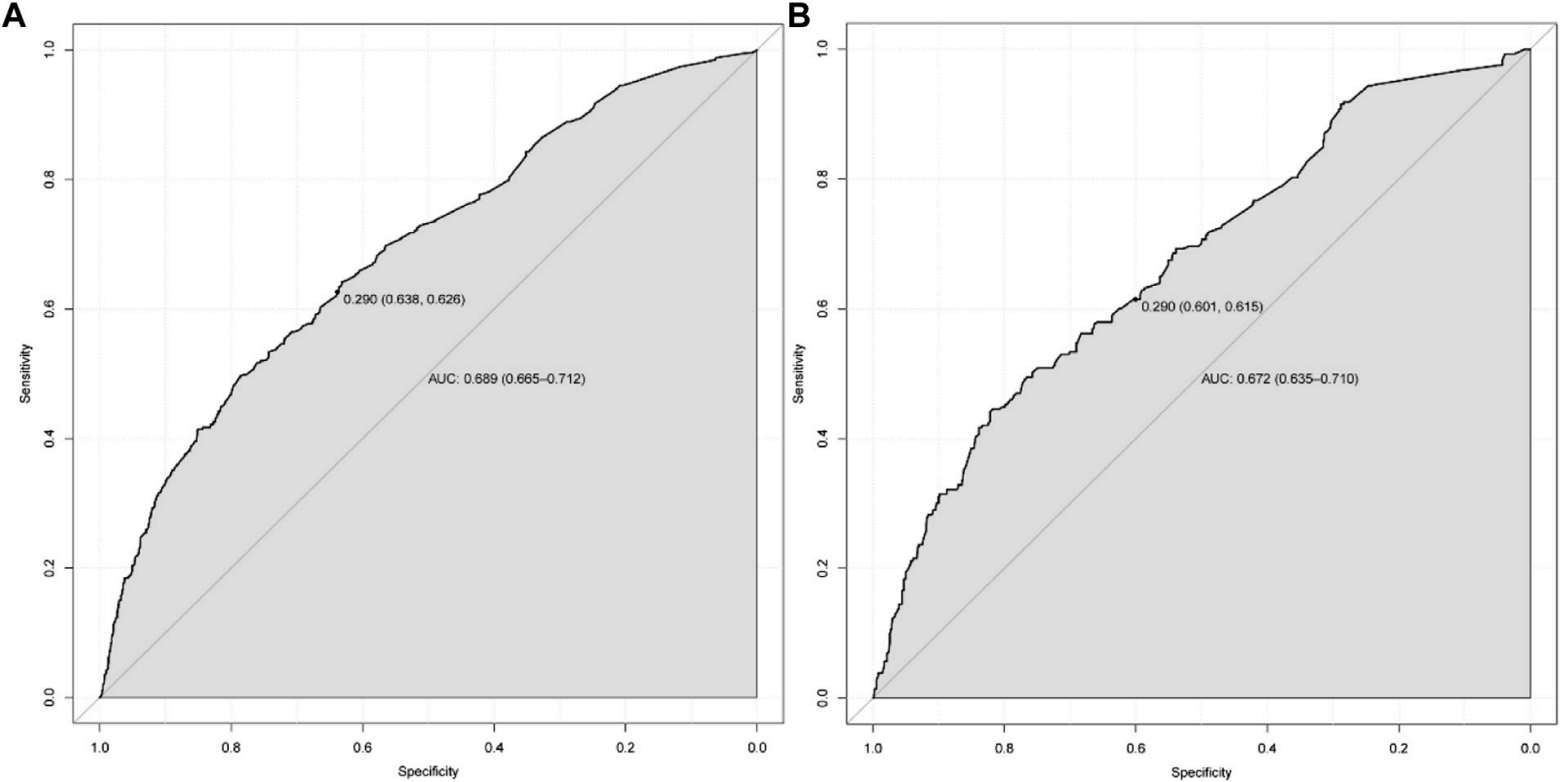
The ROC curves for the predictive model in training cohort **(A)** and validation cohort **(B)**.

**FIGURE 4:**
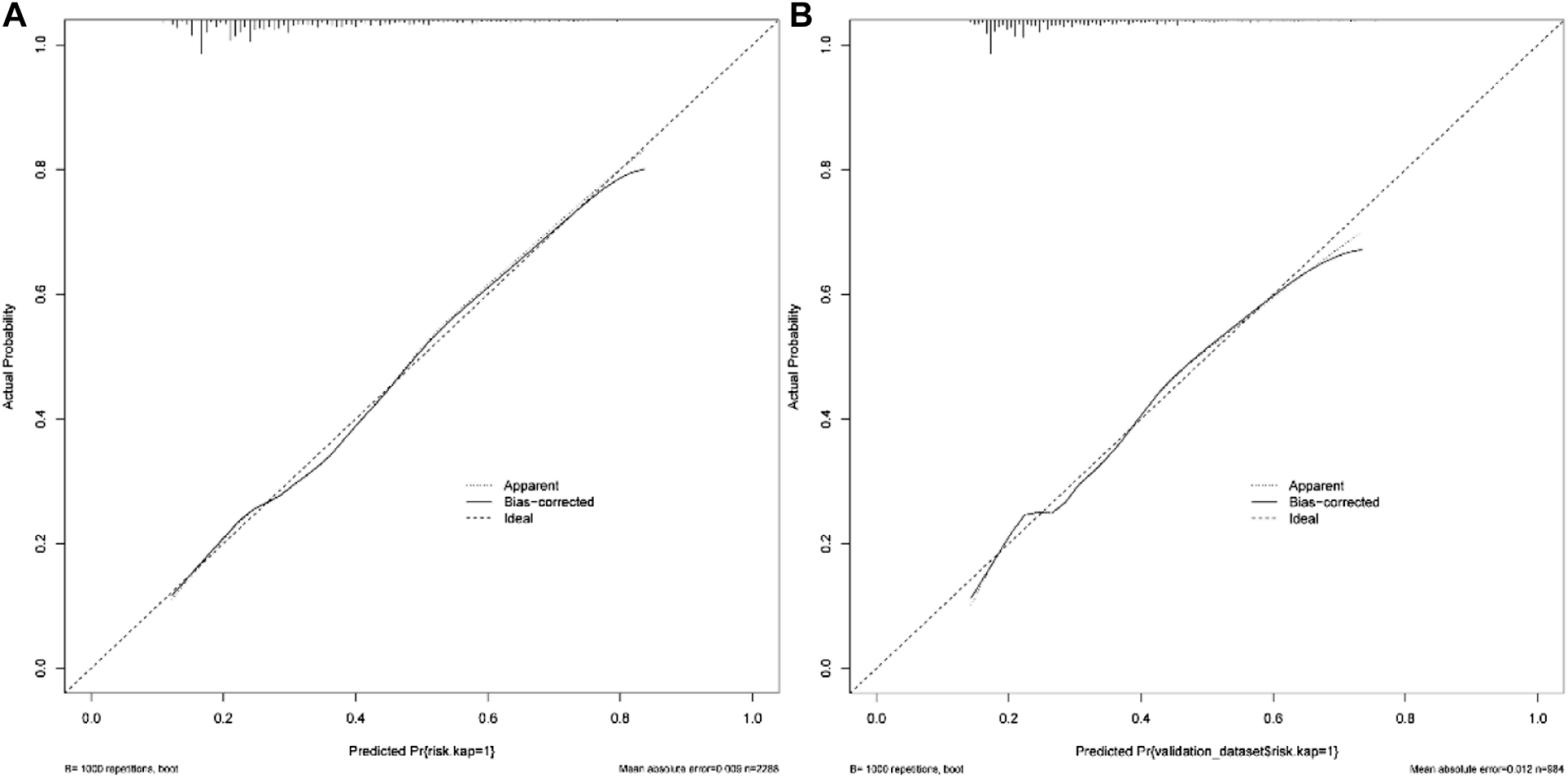
The calibration curve for the predictive medication risks in training cohort **(A)** and validation cohort **(B)**.

**FIGURE 5:**
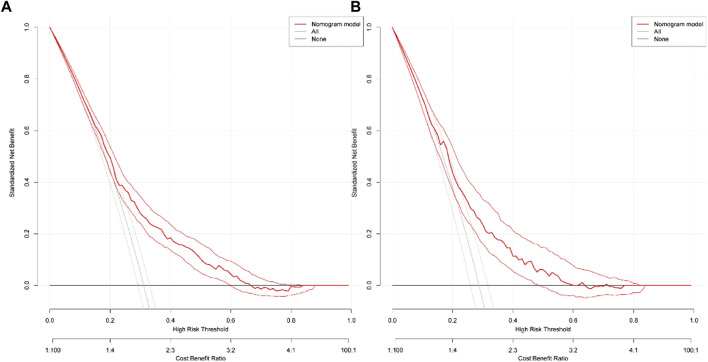
Decision curves of the nomogram predicting medication risks in training cohort **(A)** and validation cohort **(B)**.

According to the results of multiple linear regression analysis, the place of residence was also an important factor for KAP of medication, so the factor was involved in the construction of a nomogram with the multivariate analysis results. Based on the above, the nomogram for KAP risk was established (Figure 2). The score of each independent factor and the total score were identified according to the point scale at the top of the nomogram. Then, the risk of medication KAP was predicted by the total score and the point scale at the bottom of the nomogram. The predictive model showed a good ability to discriminate, as evidenced by the ROC curve with an AUC of 0.689 (95% CI = 0.665–0.712) in the training cohort and an AUC of 0.672 (95% CI = 0.635–0.710) in the validation cohort (Figure 3A, B). The calibration curve showed good agreement between the actual survey values and the model predictions for both training and validation cohorts used to predict risk of mediation KAP (Figures 4A,B). The DCA was used to evaluate the clinical utility and benefit of the risk of mediation nomogram to further assess its potential application worth. Both training and validation cohorts could receive more clinical net benefits from these results (Figure 5, B). The plot demonstrated that model-based decisions have more net benefits than non-intervention or intervention for a predicted probability threshold between 1% and 66%.

## 4 Discussion

We conducted a large survey on medication KAP among residents across the province in this paper, aiming to understand their drug use, cognition, and safety awareness. We selected 3,272 residents aged 19 or above from various regions of the province as survey subjects by random sampling. The results showed that the drug KAP level of residents in our province was generally moderate, with some misconceptions and bad habits, such as the use of antibiotics, drug storage, and disposal of expired drugs. Therefore, it is still necessary to strengthen drug education and guidance for residents and improve their ability to use drugs rationally and their self-protection awareness.

Based on this survey, linear regression was used to understand the factors affecting residents’ drug KAP scores, and high-risk groups were identified. For drug knowledge score, it was negatively correlated with sex, but positively correlated with residence, education level, and employment status. Women’s knowledge score was lower than that of men, indicating that women had better drug cognition than men. This is consistent with previous studies (Islam et al., 2020), possibly because women are responsible for taking care of children and the older adult in the family, pay more attention to health information, and are more interested in obtaining drugs (Liu et al., 2021). Residents living in rural areas had higher knowledge score than those living in urban areas, indicating that urban residents’ drug cognition was better than that of rural residents. This might be because there are more ways to obtain drug knowledge in urban areas (Xiang et al., 2010). Education level was closely related to knowledge scores. Lower education levels were associated with higher risks of medication errors. This was in accordance with the results of a study on medication KAP among Haikou residents (Wu et al., 2021). The knowledge score of employment status showed that retired or unemployed residents had higher score than currently employed ones, indicating that working residents had the best drug awareness. This might be because working residents had more communication and a wider range of ways to obtain drug knowledge (Lombardo and Cosentino, 2016). For medication attitude scores, sex had a positive correlation with medication attitudes. Women’s attitude scores were higher than men’s, and women were more willing to participate in medication education activities and related training. For medication practices, sex, Health insurance coverage, and educational background were all important influencing factors. Women were still better than men in medication practices. Basic social medical insurance was superior to other insurance. Theoretically, the higher the education level, the better the drug behavior. However, we found the opposite result in good behavior. This might be because the group with low education had more “unclear” situations in the questionnaire, which increased the weight of the impact on the score.

We developed and validated a nomogram to predict the risk of medication in residents, which can be used to guide clinical decision-making. Our results indicate that the nomogram has good discrimination, calibration, and some net clinical benefit. The nomogram contains six predictors: sex, monthly income, place of residence, insurance status, education level, and employment status. Among these predictors, female had a significantly lower risk of medication errors than male (R = 0.64, *p* = 0.00), indicating that the overall risk of medication errors was higher in the male group, and subsequent attention should be paid to this group. Compared with low-income people, high-income people have a lower risk of medication errors, and those with monthly income below 1,000 (reference) or 1,000–2000 (OR = 0.90, *p* = 0.75) had a greater risk of medication errors, and they should be the focus of pharmacy interventions. In terms of medical security status, commercial insurance (OR = 2.16, *p* = 0.00), out-of-pocket medical care (OR = 1.59, *p* = 0.02) and others (OR = 2.62, *p* = 0.00) had a higher risk of medication errors than basic social medical insurance, and these groups should be targeted. In terms of education level, compared with graduate students, middle school (OR = 1.68, *p* = 0.04) had a higher risk of medication errors, which verified the importance of education to some extent. In terms of work status, compared with current employed, retired (OR = 1.94, *p* = 0.00), unemployed or jobless (OR = 1.49, *p* = 0.02) had a higher risk of medication errors, which may be because most of the working people were in their prime age, had strong interest and ability to accept new things, and they can communicate and discuss with more people in the daily environment, and have a higher degree of awareness than retirees and unemployed people. In terms of occupation, compared with factory workers, healthcare workers (OR = 0.42, *p* = 0.00) and students (OR = 0.52, *p* = 0.02) have less risk in terms of medication errors, while company employees (OR = 0.98, *p* = 0.97), government cadres (OR = 1.12, *p* = 0.75), business managers (OR = 0.98, *p* = 0.97), and Others (OR = 1.03, *p* = 0.97) had a higher risk of medication errors and should be paid more attention to these groups. Finally, we found that male, low-income groups, commercial insurance and self-paid medical care groups, groups with lower education levels, factory workers, company employees, government officials, and businessmen were people with a high incidence of drug risk. In the follow-up work, targeted medication education activities should be carried out for such high-risk groups.

The questionnaire method is a popular tool for gathering data due to its direct approach in obtaining information. However, the questionnaire still has certain limitations. Due to the large number of questions and the extensive range of dimensions involved, some respondents lacked the patience to complete the questionnaire, resulting in only 5.07% of participants being aged 65 and above. In a major agricultural and mining province, rural residents made up just 24.63% of the survey, suggesting that the sample coverage should be enhanced. Certain sections of the questionnaire are highly specialized and may be beyond the comprehension of respondents, potentially compromising the accuracy of patient reports on adherence to treatment regimens, which is often linked to literacy levels (Lee et al., 2017). Questionnaires are known to be less sensitive (Basu, 2021), and patients may be prone to social desirability bias, which can affect the accuracy of survey data. The fixed format of the questionnaire might restrict the opportunity for respondents to provide deeper insights. In this study, for some patients with low education levels, face-to-face interview surveys are conducted to gather samples. For those who find it inconvenient to complete the questionnaire themselves, responses are dictated to the investigator and collected on the spot, enhancing the diversity and representativeness of the sample, but this is not sufficient. Therefore, in subsequent studies, we will complement the questionnaire data with qualitative research methods or design a more flexible questionnaire format to allow for more free-form feedback. Additionally, pre-testing the questionnaire to assess the validity of the questions and including appropriate open-ended questions in the design are also effective strategies to improve data quality. A more strategic distribution approach will ensure the questionnaire reaches a diverse group of respondents, making the data more comprehensive, reliable, and enhancing the validity and universality of the research.

Logistic regression is characterized by its strong interpretive ability and its effectiveness in handling binary classification problems. Based on the KAP scores, this paper categorizes residents’ drug risk into two groups: “normal” and “risk”. It identifies risk factors affecting residents’ drug use through logistic regression analysis. For the first time, a drug risk assessment model for residents is established using a nomogram, providing a simple and effective prediction tool for the majority of residents. The model’s differentiation, predictive accuracy, and clinical applicability are evaluated through ROC curves, calibration plots, and DCA. In this study, the AUC area, sensitivity and specificity of the model at the optimal threshold still did not reach the ideal results. In subsequent research, we aim to enhance both the quantity and quality of the questionnaire by optimizing its design, selecting suitable delivery channels, and strengthening its logical structure. Additionally, we will explore various algorithms and model parameters to improve the model’s predictive accuracy and discriminatory power, thereby better serving our research objectives.

In the follow-up work, we will develop corresponding drug education materials for common medication misconceptions in the questionnaire, such as disposal of expired drugs (Alnahas et al., 2020). We will also cater to the needs of special groups such as men, low-income and rural residents, and conduct personalized drug science popularization activities. For example, for the older adult, we will offer medication training lectures, current medication consultation activities, etc. Furthermore, we will implement the “Internet + pharmacy” service model (Baldoni et al., 2019), use WeChat public accounts (Liu et al., 2023), hospital mini programs and other Internet platforms to provide general pharmacy services and conduct medication education services online (Selby et al., 2015). Finally, we will organize pharmacist community activities, and relevant pharmacists will sign contracts with community hospitals and families to assist community doctors and patients to familiarize themselves with medication-related knowledge and enhance public awareness and education, strengthening drug regulation and supervision, promoting rational medication use and consultation, and reducing antibiotic misuse or abuse.

## 5 Conclusion

In summary, a new monogram was established to predict the medication risks for residents based on a large, province-wide KAP survey. The tool can help residents assess their medication risk and report their medication knowledge and practices to doctors, who can then implement appropriate interventions to reduce medication adverse effects.

## Data Availability

The original contributions presented in the study are included in the article/Supplementary Material, further inquiries can be directed to the corresponding authors.
