# Supplementary material for: Development and validation of a nomogram to predict medication risk based on a knowledge, attitude and practice (KAP) survey of residents in Shanxi Province, China
Source: Front Pharmacol. 2024 Apr 17;15:1302274. doi: 10.3389/fphar.2024.1302274 (PMC11070833; doi:10.3389/fphar.2024.1302274)
Supplement: Supplementary file 1 [file Table1.DOCX]

**KAP questionnaire on the risk of residential medication behavior**

1. Your gender

□ Male

□ Female

1. Your age

□ 19-34 years old

□ 35-49 years old

□ 50-64 years old

□ Over 65 years old

1. Your monthly income

□ Below 1000 yuan

□ 1000-2000 yuan

□ 2000-4000 yuan

□ 4000-6000 yuan

□ More than 6000 yuan

4. Your place of residence

□ Urban areas

□ Rural areas

5. Your medical insurance status

□ Basic social medical insurance

□ Commercial insurance

□ Out-of-pocket medical care

□ Publicly-funded medical care

□ others

1. Your level of education

□ Graduate student

□ Bachelor

□ Junior college

□ Technical secondary or high school

□ Middle school

□ Primary school

1. Your working conditions

□ Currently employed

□ Retired

□ Unemployed or jobless

1. Your profession

□ Factory workers

□ Company employees

□ Government cadres

□ Health care workers

□ Teacher

□ Business management

□ Freelancers

□ Student

□ others

a. Each of the following questions contains your views and opinions on drugs and their use, quantifying their approval by numbers, for example: 1 represents strong disapproval; 2 deputies disagree, but not to a greater extent; And so on; 5 means that you strongly agree, the higher the number, the higher the degree of approval, and each question item needs to be given a corresponding number that you agree with. Do you agree with these views?

1 Strongly Disagree 2 Disagree 3 General 4 Agree 5 Strongly Agree 6 Not Clear

01. If you are sick, you should try to get injections and hang water

02. Injections, hanging water and other injection drugs are safer than oral drugs

03. The more expensive the drug, the safer

04. The more expensive the drug, the more effective it is

05. The more varieties of medication, the better the curative effect

06. The longer the course of medication, the better

07. If you feel slightly unwell, you should take medication immediately

08. Health products are medicines

09. Eating health products can reduce the amount of medicines

10. Oral medicine can be taken with milk, coffee or drinks

11. Nutraceuticals can be taken at the same time as medicines

12. When infusion in the hospital, you can shorten the infusion time by yourself

13. Antibacterial drugs can cure any kind of cold and fever

14. Antibacterial drugs can kill any bacteria and viruses

15. As long as you do not abuse antimicrobials, you will not develop drug resistance

16. The more types of antibacterial drugs taken at the same time, the faster the disease will improve

17. Antibacterial drugs are anti-inflammatory drugs

18. There will be no adverse effects when taking over-the-counter medications

19. Inexhaustible medicines should be stored in the refrigerator as much as possible

20. When you feel that your symptoms are relieved, you can stop the drug

21. When you feel that your symptoms are relieved, you can reduce the dose of medicine

22. When the self-feeling symptoms are aggravated, the dose of the drug can be increased

23. When the self-feeling symptoms are aggravated, the type of drug can be changed

24. The efficacy and safety of new drugs are definitely better than old drugs

25. When people around you have a cold, fever or other diseases, you can take antibacterial drugs for prevention

26. When purchasing drugs, you should judge the quality of drugs based on brand and reputation

27. All medicines can be stored at room temperature

28. When buying medicine, the price does not matter, the key is good efficacy

b. The following describes some of the behaviors during your use of drugs: Please circle the frequency and frequency of these behaviors (quantitative assignment is the same as above).

1 Never 2 Occasionally 3 Sometimes 4 Often 5 Always 6 Not Clear

29. Purchase medicines on their own basis based on their own experience or as advertised

30. When seeing a doctor, ask the doctor by name to prescribe a certain medicine

31. Follow the advice of the salesperson when buying medicines at the pharmacy

32. Go to the pharmacy with a doctor's prescription to buy prescription drugs

33. Conduct regular inspections of medicines stored at home

34. Keep medicines within the reach of children

35. Observe the conditions under which the drug is stored in the instructions

36. Look at the side effects of the drug and how they behave after they appear

37. Know the contraindications when using a drug (drinking, driving, etc.)

38. Observe the expiration date of the drug before taking the drug

39. Check the drug approval number on the drug packaging before taking the medicine

40. Discard expired medicines at home in the trash

41. Forgetting to use medicines while taking them

42. The child refuses to take the medicine and pinches his nose to fill him with medicine

43. Taking medicines that have expired

44. Take the medicine at the same time as milk, tea or coffee

45. When taking medication, you will consider your physical condition every time

46. In order to increase the efficacy or increase the dose without authorization when the symptoms are aggravated

47. Not paying attention to the time interval between taking the drug

48. After taking the medicine for a few days, if you feel that it has no effect, change the drug without authorization

1. After the condition improves, reduce the dose or stop the drug on your own
2. While taking Western medicine, choose Chinese medicine without authorization
3. Break or crush the tablets before eating
4. Without the knowledge of your doctor or pharmacist, you will mix several drugs

c. The following describes medication knowledge lectures or medication education activities that you may have been exposed to, please circle how often you actually come into contact with or carry out these activities. (Quantitative assignment as above).

1 Never 2 Occasionally 3 Sometimes 4 Often 5 Always 6 Not Clear

53. Listen to a lecture on the knowledge of rational drug use in the community where I live

54. Listen to lectures on rational drug use in hospitals or community health service centers

55. Read publicity materials on rational drug use distributed by community neighborhood committees

56. Pharmacist community or street counseling services

57. Read publicity materials on rational drug use issued by hospitals or community health service centers

58. Read publicity materials on rational drug use in street windows

d. If you have been exposed to the above forms of educational activities on rational drug use, what is your view of the need to carry out these activities? (Quantitative assignment as above).

1 Extremely unnecessary 2 Not necessary 3 General 4 Necessary 5 Extremely necessary 6 Unclear

59. Lectures on the rational use of drugs in the community

60. Lectures on rational drug use in hospitals or community health service centers

61. Community neighborhood committees distribute publicity materials on rational drug use

62. Publicity materials on rational drug use in hospitals or community health service centers

63. Publicity materials displaying knowledge on rational drug use in street windows
